# The Time Evolution of Mutual Information between Disjoint Regions in the Universe

**DOI:** 10.3390/e25071094

**Published:** 2023-07-21

**Authors:** Biswajit Pandey

**Affiliations:** Department of Physics, Visva-Bharati University, Santiniketan 731235, India; biswap@visva-bharati.ac.in

**Keywords:** mutual information, large-scale structure of the universe, evolution of the universe, cosmology

## Abstract

We study the time evolution of mutual information between mass distributions in spatially separated but casually connected regions in an expanding universe. The evolution of mutual information is primarily determined by the configuration entropy rate, which depends on the dynamics of the expansion and growth of density perturbations. The joint entropy between distributions from the two regions plays a negligible role in such evolution. Mutual information decreases with time in a matter-dominated universe, whereas it stays constant in a Λ-dominated universe. The ΛCDM model and some other models of dark energy predict a minimum in mutual information beyond which dark energy dominates the dynamics of the universe. Mutual information may have deeper connections to the dark energy and accelerated expansion of the universe.

## 1. Introduction

Entropy plays a key role in understanding a wide range of phenomena in science. It plays an important role in deciding the evolution of the universe. The universe is regarded as a dynamical system in cosmology. The dynamics of the expansion of the universe is described by Friedmann equations, which are based on Einstein’s equation of general relativity and the cosmological principle. The cosmological principle assumes that the universe is statistically homogeneous and isotropic on sufficiently large scales. The validity of this assumption is crucial to our understanding of modern cosmology. A large number of studies have been carried out to verify the cosmological principle. Studies based on various cosmological observations have found that the universe is statistically homogeneous and isotropic on scales somewhere beyond 70–150 Mpc [1,2,3,4,5,6,7,8,9,10,11,12,13]. The universe is highly inhomogeneous and anisotropic on smaller scales due to the presence of a clear hierarchy of structures, starting from planets, stars, and galaxies to groups, clusters, and superclusters. All these structures in the present universe are believed to have emerged from the growth of primordial density fluctuations seeded in the early universe. The observed CMBR temperature fluctuations of $\frac{\Delta T}{T}∼10^{-5}$ at redshift z∼1100 suggest that the universe was highly homogeneous and isotropic in the past. These tiny fluctuations are amplified by gravitational instability, producing structures over a wide range of length scales.

Our universe is known to be expanding. Recent observations have suggested that the universe is currently undergoing accelerated expansion [14,15]. Understanding the present accelerated expansion of the universe is a major unsolved problem in cosmology. The dynamics of this expansion affect the growth of inhomogeneities in the universe. Conversely, the inhomogeneities may also play an important role in the observed acceleration through their effect on the large-scale dynamics of the universe [16,17,18,19,20]. Reference [21] suggested that the observed acceleration of the universe is consistent with the second law of thermodynamics, and the entropy of the universe in the ΛCDM model tends toward a finite maximum. Interestingly, alternative models, such as non-singular bouncing universes, modified gravity theories, and phantom fields, do not lead to a state of maximum entropy [22,23,24]. The continuous dissipation of information entropy of matter distribution due to gravitational instability may also drive the accelerated expansion of the universe [25,26]. These studies suggest that the large-scale inhomogeneity and the maximum entropy production principle (MEPP) [27,28] may have important roles in the observed acceleration of the universe.

Numerous works in the literature have pointed to the existence of very large-scale structures in the universe. The ‘Sloan Great Wall’ in the nearby universe extends to scales greater than 400 Mpc [29]. Reference [30] found a large quasar group that extends to 500 h${}^{-1}$ Mpc at z∼1.3. More recently, Reference [31] reported the discovery of an enormously large giant arc at z∼0.8 that spans ∼1 Gpc. Reference [32] found correlated orientations of the axes of large quasar groups on Gpc scales. A supervoid of a diameter of ∼600 h${}^{-1}$ Mpc detected by [33] indicates the possible existence of very large underdense regions in the universe. Other studies have reported the evidence of bulk flow from the analysis of Type Ia supernovae [34] and the existence of anomalously large dipoles in the distribution of quasars [35]. These findings indicate the presence large-scale inhomogeneity and anisotropy that are in apparent contradiction with the cosmological principle. However, there can always be homogeneity and isotropy on some larger scales. Thus, it is difficult to falsify the cosmological principle solely based on these observations. Further, the statistical significance of these structures are questionable [11,36,37]. In any case, these observations are interesting in their own right and require further scrutiny to arrive at a conclusion.

A wide variety of statistical tools are used to characterize the inhomogeneities in the universe. The n-point hierarchy of the correlation functions and their Fourier transforms—the polyspectra [38]—are widely used to study inhomogeneous matter distribution in cosmology. Minkowski functionals can measure the morphology of large-scale structures in the universe [39]. Kullback–Leibler relative information entropy can distinguish the local inhomogeneous mass density field from its spatial average [40,41]. Reference [42] showed that a non-negligible dynamical entanglement may arise due to mutual information between spatially separated but causally connected regions. Reference [43] used Tsallis relative entropy to calculate mutual information between spatially separated but causally connected regions of the universe. Reference [44] studied the Renyi mutual information between distant spatial regions in the vacuum state of a free scalar field. Reference [45] showed that mutual information between two spatial regions may become enhanced due to inflation. The mutual information of disjoint regions in higher dimension is discussed in [46].

In the present work, we want to calculate Shannon mutual information between disjoint but causally connected regions in an expanding universe. The spatial distributions of matter in any two distant regions may have finite mutual information due to the presence of large-scale structures and long-range correlations. We do not consider an inhomogeneous universe for our current analysis. We consider a homogeneous and isotropic universe and study the time evolution of mutual information between any two distant regions. It would be interesting to investigate the role of different constituents of the universe in the time evolution of mutual information between distant regions.

The plan of our work is as follows. We describe the time evolution of the mutual information in an expanding universe in Section 2, describe the results in Section 3 and present our conclusions in Section 4.

## 2. Mutual Information and Its Time Evolution

The configuration entropy associated with matter distribution over a significantly large volume, *V*, of the universe is defined as [25]

(1) $$S\left(t\right)=-\int \rho \left(\vec{r},t\right)\;\log\rho \left(\vec{r},t\right)\;dV$$

The volume, *V*, is subdivided into a number of subvolumes, dV, and the density, $\rho (\vec{r},t)$, is measured within each of them. Here, *r* describes the comoving coordinate associated with the center of the subvolumes, and $\rho (\vec{r},t)$ refers to the matter density in the subvolumes. The density, $\rho (\vec{r},t)$, is directly related to the probability of finding a mass element within a subvolume.

**Figure 1:**
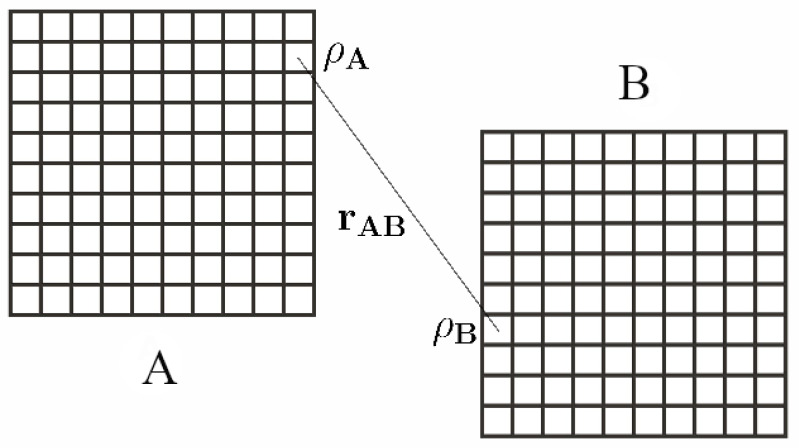
This figure shows two large identical volumes, *A* and *B*, divided into an equal number of subvolumes. Here, $\rho_{A}$ and $\rho_{B}$ refer to the density within any two subvolumes at a given instant, *t*, and $r_{AB}$ is the radial separation between the two subvolumes under consideration. We consider *A* and *B* to be causally connected.

Let us consider two large identical volumes, *V*, separated by a large distance. We label these two volumes as *A* and *B* (Figure 1). The two regions, *A* and *B*, are causally connected. The configuration entropy of the two regions, *A* and *B*, can be written as

(2) $$S_{A}\left(t\right)=-\int \rho_{A}\left(\vec{r},t\right)\;\log\rho_{A}\left(\vec{r},t\right)\;dV_{A}$$

and

(3) $$S_{B}\left(t\right)=-\int \rho_{B}\left(\vec{r},t\right)\;\log\rho_{B}\left(\vec{r},t\right)\;dVB$$

We consider *A* and *B* to be significantly large volumes so that the universe can be treated as statistically homogeneous and isotropic on those scales. We are only interested in scales where one can safely use linear perturbation theory to describe the evolution of configuration entropy in these regions.

One can define the mutual information between the mass distributions within the two regions, *A* and *B*, as

(4) $$I_{AB}\left(t\right)=\int \int \rho_{AB}\left(\vec{r_{1}},t\;;\vec{r_{2}},t\right)\;\log\frac{\rho_{AB}\left(\vec{r_{1}},t\;;\vec{r_{2}},t\right)}{\rho_{A}\left(\vec{r_{1}},t\right)\;\rho_{B}\left(\vec{r_{2}},t\right)}\;dV_{A}\;dV_{B}$$

where $\rho_{AB}\left(\vec{r_{1}},t\;;\vec{r_{2}},t\right)$ is the joint density distributions in the two volumes. This provides the joint probability of finding a mass element within each of the two subvolumes. One can simplify Equation (4) to write the mutual information between the two regions as

(5) $$I_{AB}\left(t\right)=S_{A}\left(t\right)+S_{B}\left(t\right)-S_{AB}\left(t\right)$$

where the joint entropy $S_{AB}\left(t\right)$ can be expressed as

(6) $$\begin{array}{cc} S_{AB}\left(t\right) & =-\int \int \rho_{AB}\left(\vec{r_{1}},t\;;\vec{r_{2}},t\right)\;\log\rho_{AB}\left(\vec{r_{1}},t\;;\vec{r_{2}},t\right)\;dV_{A}\;dV_{B}\\ \; & =-\int \int {\bar{\rho }}_{A}\left(t\right)\;{\bar{\rho }}_{B}\left(t\right)\;\left[1+\xi \left({\vec{r}}_{AB},t\right)\right]\;\log\left[{\bar{\rho }}_{A}\left(t\right)\;{\bar{\rho }}_{B}\left(t\right)\;\left(1+\xi ({\vec{r}}_{AB},t)\right)\right]\;dV_{A}\;dV_{B} \end{array}$$

where $\xi ({\vec{r}}_{AB},t)$ is the two-point correlation function, and ${\bar{\rho }}_{A}\left(t\right)$ and ${\bar{\rho }}_{B}\left(t\right)$ are the mean densities in the two regions, *A* and *B*. The separation vector between the center of the two subvolumes is ${\vec{r}}_{AB}=\vec{r_{2}}-\vec{r_{1}}$. The two-point correlation function, $\xi ({\vec{r}}_{AB},t)$, provides the excess probability of finding two mass elements separated by ${\vec{r}}_{AB}$. Mutual information, $I_{AB}\left(t\right)$, quantifies the reduction in uncertainty in mass distribution within one volume, given that we have complete knowledge of the mass distribution in the other. In other words, it quantifies the expected gain in information about mass distribution in one volume when the other volume is observed.

We assume that the matter distribution in the universe is homogeneous and isotropic on a scale of $V^{\frac{1}{3}}$. This allows us to write ${\bar{\rho }}_{A}\left(t\right)\approx {\bar{\rho }}_{B}\left(t\right)=\bar{\rho }\left(t\right)$, $S_{A}\left(t\right)\approx S_{B}\left(t\right)=S\left(t\right)$, $\xi \left({\vec{r}}_{AB},t\right)=\xi (|{\vec{r}}_{2}-{\vec{r}}_{1}\left|,t\right)=\xi \left(r_{AB},t\right)$, and $\frac{dS_{A}\left(t\right)}{dt}\approx \frac{dS_{B}\left(t\right)}{dt}=\frac{dS(t)}{dt}$.

The mean density, $\bar{\rho }\left(t\right)$, and the two-point correlation function, $\xi (r_{AB},t)$, would evolve differently in different cosmological models. The mutual information between the two regions and its time evolution would, thus, depend on the cosmological model.

## 3. Results

### 3.1. Mutual Information in a Matter-Dominated Universe

We would like to calculate the mutual information between the mass distributions in the two regions, *A* and *B*, in a matter-dominated universe ($\Omega_{m}=1$). The average density in a matter-dominated universe is $\bar{\rho }\left(t\right)=\frac{1}{6\;\pi \;G\;t^{2}}$, and the growing mode of density perturbations is $D\left(t\right)\propto t^{\frac{2}{3}}$. In a linear regime, the time evolution of the two-point correlation function can be described as $\xi \left(r_{AB},t\right)=D^{2}\left(t\right)\;\xi \left(r_{AB}\right)$. Here, the proportionality constant is absorbed in $\xi (r_{AB})$.

Reference [25] showed that the configuration entropy rate in a matter-dominated universe is always negative, i.e., $\frac{dS(t)}{dt}<0$. Let us write $\frac{dS(t)}{dt}=-f\left(t\right)$.

The time evolution of mutual information between the regions, *A* and *B*, can be written from Equation (5) as

(7) $$\frac{dI_{AB}\left(t\right)}{dt}=-2\frac{dS(t)}{dt}+\frac{d}{dt}\int \int \left[{\bar{\rho }}^{2}\left(t\right)\;\left(1+D^{2}\left(t\right)\;\xi \left(r_{AB}\right)\right)\right]\;\log\left[{\bar{\rho }}^{2}\left(t\right)\;\left(1+D^{2}\left(t\right)\;\xi \left(r_{AB}\right)\right)\right]\;dV_{A}\;dV_{B}$$

Simplifying Equation (7), we obtain,

(8) $$\frac{dI_{AB}\left(t\right)}{dt}=-2f\left(t\right)+I_{1}+I_{2}+I_{3}+I_{4}+I_{5}$$

where

(9) $$I_{1}=\frac{2}{9\;\pi^{2}\;G^{2}}\;\log\left(6\pi G\right)\int \int \left(t^{-5}+\frac{2}{3}\;t^{-\frac{11}{3}}\;\xi \left(r_{AB}\right)\right)\;dV_{A}\;dV_{B}$$

(10) $$I_{2}=-\frac{1}{9\pi^{2}G^{2}}\;\int \int t^{-5}\left(1-4\log t\right)\;dV_{A}\;dV_{B}$$

(11) $$I_{3}=-\frac{1}{9\pi^{2}G^{2}}\;\int \int \xi \left(r_{AB}\right)\;t^{-\frac{11}{3}}\left(1-\frac{8}{3}\;\log t\right)\;dV_{A}\;dV_{B}$$

(12) $$I_{4}=-\frac{1}{9\pi^{2}G^{2}}\;\int \int \left(t^{-5}+\frac{2}{3}\;t^{-\frac{11}{3}}\;\xi \left(r_{AB}\right)\right)\;\log\left(1+t^{\frac{4}{3}}\;\xi \left(r_{AB}\right)\right)\;dV_{A}\;dV_{B}$$

and

(13) $$I_{5}=\frac{1}{27\pi^{2}G^{2}}\;\int \int \left(t^{-4}+t^{-\frac{8}{3}}\;\xi \left(r_{AB}\right)\right)\;\frac{t^{\frac{1}{3}}\;\xi \left(r_{AB}\right)}{1+t^{\frac{4}{3}}\;\xi \left(r_{AB}\right)}\;dV_{A}\;dV_{B}$$

The integrals in the expressions of $I_{1},I_{2},I_{3},I_{4}$, and $I_{5}$ can not be simplified further due to the lack of symmetry. However, one can easily analyze the time dependence of these expressions. Observations show that the galaxy two-point correlation function has a nearly universal dependence on pair separation, *r*, as $\xi \left(r\right)∼r^{-1.8}$. The terms involving $\xi (r_{AB})$ in these expressions will have a smaller magnitude. Equations (9)–(13) have strong time dependence. $I_{2}$ and $I_{3}$ become positive for larger values of time. Only $I_{4}$ remains negative at all time. The sum $I=(I_{1}+I_{2}+I_{3}+I_{4}+I_{5})$ is positive but decays toward zero with increasing time. The term f(t) in Equation (8) has a much weaker time dependence as compared to *I* [25]. This leads to $\frac{dI_{AB}\left(t\right)}{dt}<0$, which implies that the mutual information between two independent regions decreases with time in a matter-dominated universe.

It may be noted that Equation (8) depends on the size of the regions, *A* and *B*, and the separation between them. The integrals in Equation (8) would be carried out over different volumes when there is a change in the size of the two regions. The separations between the different pairs of subvolumes would change with the distance between the two regions. The integrals in Equation (8) will have different values since the two-point correlation function changes with the separation. If the two regions are separated by a very large distance compared to the dimensions of the two regions then the integrals in Equation (8) would lose their physical relevance.

### 3.2. Mutual Information in a Λ-Dominated Universe

Here, we would like to calculate $\frac{dI_{AB}\left(t\right)}{dt}$ in a Λ-dominated universe. We have $\bar{\rho }\left(t\right)=\bar{\rho }=$ constant, and the growing mode of density perturbations is D(t)=k= constant in an $\Omega_{\Lambda }=1$ universe. The time evolution of mutual information in such a universe can be expressed as

(14) $$\frac{dI_{AB}\left(t\right)}{dt}=-2\frac{dS(t)}{dt}+\frac{d}{dt}\int \int \left[{\bar{\rho }}^{2}\;\left(1+k^{2}\;\xi \left(r_{AB}\right)\right)\right]\;\log\left[{\bar{\rho }}^{2}\;\left(1+k^{2}\;\xi \left(r_{AB}\right)\right)\right]\;dV_{A}\;dV_{B}$$

Reference [25] showed that $\frac{dS}{dt}=0$ in a Λ-dominated universe. Thus, we have $\frac{dI_{AB}\left(t\right)}{dt}=0$. Clearly, $I_{AB}=$ constant in a Λ-dominated universe. There would be constant mutual information between the regions, *A* and *B*, at all times, in such a universe.

### 3.3. Mutual Information in the ΛCDM Model, the Dynamical Dark Energy Models, and the Holographic Dark Energy Models

It is clear that the joint entropy between the two regions, *A* and *B*, plays a negligible role in the time evolution of mutual information. The configuration entropy rate, $\frac{dS}{dt}$, determines the time evolution of mutual information between the two regions. The configuration entropy rates have been calculated for the ΛCDM model, different dynamical dark energy models, and holographic dark energy models in the literature [47,48,49,50]. The configuration entropy rate decreases to reach a minimum and then increases with time in all these models. However, the location and amplitude of the minimum depend on the models. The location of the minimum precisely indicates the epoch of dark energy domination predicted by the relevant model. The average density will fall faster in such models as compared to a matter-dominated universe. Therefore, the joint entropy term would contribute negligibly to the evolution of mutual information in all these models. The time evolution of mutual information in a given model will be, thus, entirely determined by the behavior of the configuration entropy rate in that model. The mutual information between *A* and *B* in all these models would initially decrease with time and eventually hit a minimum. The mutual information would increase after this minimum once the dark energy starts to dominate the dynamics of the universe.

## 4. Conclusions

We analyzed the time evolution of mutual information between disjoint regions of the universe in different cosmological models. Mutual information here quantifies a reduction in uncertainty in the knowledge of matter distribution in one region, given that we have complete knowledge of it in the other region. In other words, mutual information provides some knowledge about matter distribution in one region provided we have the complete knowledge of matter distribution in the another region. Zero mutual information indicates that mass distribution in the two regions are statistically independent. We did not separately calculate mutual information between the two disjoint regions but obtained an expression for its time evolution from the definition. It may be noted that mutual information is positive or zero by definition. It can not assume negative values. However, the rate of change in mutual information can be negative, positive, or zero.

The time evolution of mutual information between disjoint regions of the universe is primarily determined by the dynamics of the expansion and growth rate of density perturbations. We found that mutual information decreases continuously in a purely matter-dominated universe, whereas it stays constant in a purely Λ-dominated universe. Thus, disjoint regions become statistically independent in a matter-dominated universe, whereas they remain entangled forever in a Λ-dominated universe. Mutual information decreases to reach a minimum and then increases with time in the ΛCDM model, dynamical dark energy models, and holographic dark energy models. Clearly, the time evolution of mutual information is governed by the changes in the configuration entropy of matter distribution in the universe. The change in joint entropy between mass distributions in the two regions does not contribute significantly to this evolution.

The two regions *A* and *B* are causally connected. However, they may be separated by a large distance. In reality, one cannot measure mass distributions in the two volumes simultaneously. One can infer some information about the mass distribution in one volume while observing the other. This information corresponds to the same cosmic time. It is worthwhile to mention here that mutual information between the two causally connected regions may also introduce a non-negligible dynamical entanglement [42]. The effect of such dynamical entanglement is not considered in the present work. Further, one can also consider the contributions from higher-order correlations. A non-zero three-point correlation function would modify the joint probabilities in Equation (4). We plan to address these issues in future works. It would be also interesting to study the evolution of mutual information in inhomogeneous cosmological models. The presence of long-range correlations in mass distributions can significantly modify mutual information and its evolution.

The continuous dissipation of configuration entropy during a matter-dominated era demands enormous entropy production that can counterbalance this loss and maximize entropy [25]. The accelerated expansion of the universe provides an avenue for maximum entropy production in accordance with the second law of thermodynamics. It is interesting to note that the evolution of mutual information is strongly sensitive to the cosmological constant or dark energy. This implies that mutual information may have deeper connections to dark energy and the accelerated expansion of the universe.

## Data Availability

Not applicable.

## Abbreviations

The following abbreviations are used in this manuscript:

CMBR	Cosmic Microwave Background Radiation
ΛCDM	Lambda Cold Dark Matter
MEPP	Maximum Entropy Production Principle
